# Tailoring Energy Transfer in Mixed Eu/Tb Metal–Organic Frameworks for Ratiometric Temperature Sensing

**DOI:** 10.3390/molecules29163914

**Published:** 2024-08-19

**Authors:** Hui Tang, Siyuan Cheng, Zhihui Zhang, Mingyang He, Junfeng Qian, Liang Li

**Affiliations:** 1 Jiangsu Key Laboratory of Advanced Catalytic Materials and Technology, Changzhou University, Changzhou 213164, China; tanghui090920@163.com (H.T.); csy6936@163.com (S.C.); hmy@cczu.edu.cn (M.H.); qianjunfeng@cczu.edu.cn (J.Q.); 2 Xinjiang Key Laboratory for Luminescence Minerals and Optical Functional Materials, School of Physics and Electronic Engineering, Xinjiang Normal University, Urumqi 830054, China

**Keywords:** luminescent thermometers, metal–organic frameworks, energy transfer, thermal sensitivity, lanthanides

## Abstract

Eu/Tb metal–organic frameworks (Eu/Tb-MOFs), exhibiting Eu^3+^ and Tb^3+^ emissions, stand out as some of the most fascinating luminescent thermometers. As the relative thermal sensitivity model is limited to its lack of precision for fitting ratio of Eu^3+^ and Tb^3+^ emissions, accurately predicting the sensing performance of Eu/Tb-MOFs remains a significant challenge. Herein, we report a series of luminescent Eu/Tb-MOF thermometers, Eu_x_Tb_1−x_L, with excellent thermal sensitivity around physiological levels, achieved through the tuning energy transfer from ligands to Eu^3+^ and Tb^3+^ and between the Ln ions. It was found that the singlet lowest-energy excited state (*S*_1_) of the ligand and the higher triplet energy level (*T_n_*) are crucial in the energy transfer processes of ligand→Tb^3+^ and ligand→Eu^3+^. This enables Eu_x_Tb_1−x_L to serve as an effective platform for exploring the impact of these energy transfer processes on the temperature-sensing properties of luminescent Eu/Tb-MOF thermometers. The relative thermal sensitivity is comparable to that of dual-center MOF-based luminescent thermometers operating at physiological levels. This study provides valuable insights into the design of new Eu/Tb thermometers and the accurate prediction of their sensing performance.

## 1. Introduction

Precision temperature sensing is vital in various fields, such as large-area temperature mapping, biology, and chemical reaction monitoring [1,2,3,4,5,6,7,8,9,10]. Lanthanide metal–organic frameworks (Ln-MOFs), crystalline porous materials constructed from lanthanide nodes and organic ligands, have garnered significant interest in luminescence thermometry [11,12,13,14,15,16,17,18]. Among the various Ln-MOF-based luminescent thermometers, Eu/Tb-MOFs [19,20,21,22,23,24,25,26,27,28,29,30,31] are frequently used to determine the temperature based on the ratio between the integrated intensities of the ^5^D_4_→^7^F_5_ and ^5^D_0_→^7^F_2_ transitions of Tb^3+^ and Eu^3+^, respectively. Due to the diverse organic ligands available, many Eu/Tb-MOFs with high relative thermal sensitivity have been reported, functioning across temperature ranges from cryogenic to physiological. Over the past decade, some efforts have been dedicated to fine-tuning the temperature-sensing properties of Eu/Tb-MOFs. However, the observed thermal-responsive luminescence involves complex energy transfer processes between ligands and Tb^3+^/Eu^3+^ ions, as well as from Tb^3+^ to Eu^3+^. Consequently, a general model with which to precisely predict the temperature-sensing properties of these thermometers has not yet been proposed. As a result, many exceptional Eu/Tb-MOF thermometers are still being discovered serendipitously, and rationalizing their relative thermal sensitivity and operating temperature range remains a significant challenge in the field.

Given that excited-state calculations of Eu/Tb-MOFs are time-consuming, the Mott–Seitz model is frequently employed as a rapid experimental approach to rationalize the thermal quenching of luminescence in these materials [32,33,34]. However, a significant limitation of the Mott–Seitz model is its lack of precision when used to fit the ratio between the integrated intensities of Tb^3+^ and Eu^3+^ emissions in Eu/Tb materials. This shortcoming is often attributed to the empirical observation [35,36,37,38,39,40] that Tb^3+^ emission is influenced by the energy difference (Δ*E_T_*) between the triplet lowest-energy excited state (*T*_1_) of the ligand and the lowest emitting levels of Tb^3+^. Conversely, the model assumes that the Eu^3+^ transition is unaffected by temperature variations. Consequently, the integrated intensity ratio is predominantly determined by Δ*E_T_*. However, this prediction is not consistently observed in practical applications. In addition to *T*_1_, higher energy levels of ligands, such as the singlet lowest-energy excited state (*S*_1_), may also play a crucial role in the energy transfer processes between the ligand and Tb^3+^/Eu^3+^ in certain Eu/Tb-MOFs. Moreover, recent findings indicate that Eu^3+^ content is another significant structural factor. Therefore, considerable efforts are still needed to explore the contributions of energy transfers between the higher energy levels of ligands and Tb^3+^/Eu^3+^, as well as the impact of Eu^3+^ content on the temperature-sensing performance of Eu/Tb-MOF thermometers.

Herein, we report the investigation of TbL and Eu_x_Tb_1−x_L (x = 0.0001, 0.0005, and 0.001). Aiming to bridge the gap between the composition of Eu/Tb-MOFs and their desired thermometric performance, we examined the temperature-dependent luminescence of TbL and Eu_x_Tb_1−x_L. This study demonstrates that energy transfer likely occurs from *S_1_* and higher triplet energy levels (*T_n_*) of the ligand to Tb^3+^ or Eu^3+^ and establishes how to optimize the thermometric performance of Eu_x_Tb_1−x_L by selecting appropriate ligands and adjusting Eu^3+^ content. The results offer valuable insights for further improvements in the rational design of Eu/Tb-MOF luminescent thermometers, enabling their effective operation across temperature ranges from cryogenic to physiological levels.

## 2. Results and Discussion

### 2.1. Structural Properties of TbL and Eu_x_Tb_1−x_L

In recent years, we have reported the structural and luminescence properties of a Tb-MOF and a series of Eu/Tb-MOFs based on a tetracarboxylic acid ([1,1:4,1-terphenyl]-2,4,4,5-tetracarboxylic acid, H_4_L) [41], referred to as TbL and Eu_x_Tb_1−x_L, respectively. Despite the triplet excited-state energy of H_4_L (20,661 cm^−1^) being very close to the ^5^D_4_ energy level of Tb^3+^ (20,500 cm^−1^), TbL emits bright green light originating from Tb^3+^ upon UV excitation. For Eu_x_Tb_1−x_L, both the ^5^D_4_→^7^F_5_ transition of Tb^3+^ and the ^5^D_0_→^7^F_2_ transition of Eu^3+^ are observed in the emission spectra. These results make Eu_x_Tb_1−x_L an excellent platform to investigate the effects of *S*_1_, *T_n_*, and Eu^3+^ content on temperature-sensing properties.

**Figure 1:**
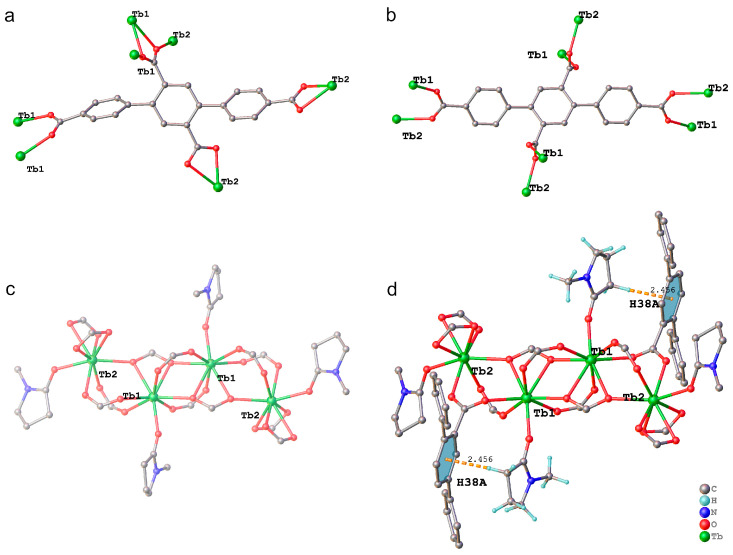
Views of (**a**) *μ*_7_ and (**b**) *μ*_8_ coordination modes of ligand. (**c**) View of Tb_4_(COO)_12_·4NMP cluster. (**d**) C-H···π hydrogen bond within crystal structure of TbL.

TbL and Eu_x_Tb_1−x_L are in soluble in water and common organic solvents such as methanol and ethanol. Figure 1 shows the coordination environment of Tb^3+^ ions, L^4−^ ligands, and supramolecular interactions within the crystal structure of TbL. The asymmetric unit of TbL includes two crystallographically unique Tb^3+^ ions (Tb1 and Tb2), two L^4−^ ligands (one of which is on the inversion center), and two coordinated NMP molecules. As shown in Figure 1a,b, there exist *μ*_7_ and *μ*_8_ coordination modes for the ligand. Two Tb1 and two Tb2 are linked by 12 carboxylates and 4 NMP to form the Tb_4_(COO)_12_·4NMP cluster (Figure 1c). Importantly, a C-H···π hydrogen bond with a 2.456 Å H···Cg length (C38-H38A→Cg8) was observed between the ligand on the inversion center and the NMP molecule coordinated with Tb1 (Figure 1d). In contrast, π-π stacking was not observed. This result indicates that Tb^3+^ emission is sensitized by excited ligand monomers. The structures of TbL and Eu_x_Tb_1−x_L were confirmed by powder X-ray diffraction (PXRD) (Figure S1), thermogravimetric analysis (TGA) (Figure S2), and Fourier transform infrared (FT-IR) spectroscopy (Figure S3). For TbL and Eu_x_Tb_1−x_L, TGA curves were very similar in the temperature range from room temperature to 723 K. At higher temperatures, the TGA curves of two compounds with x = 0.0001 and 0.0005 were different from the other ones. This is probably due to complex phase changes in combustion of the ligand molecules in the temperature range. The characteristic IR absorption bands of amide C=O vibration and sp^3^ C−H vibration in NMP are observed around 1670 and 2890 cm^−1^, respectively. The IR spectra difference, near 3500 cm^−1^, between the compounds resulted from adsorbed water on the surface of particles. These results demonstrate that the samples of TbL and Eu_x_Tb_1−x_L (x = 0.0001, 0.0005, and 0.001) are essentially pure phases.

### 2.2. Photoluminescence Properties of TbL and Eu_x_Tb_1−x_L

**Figure 2:**
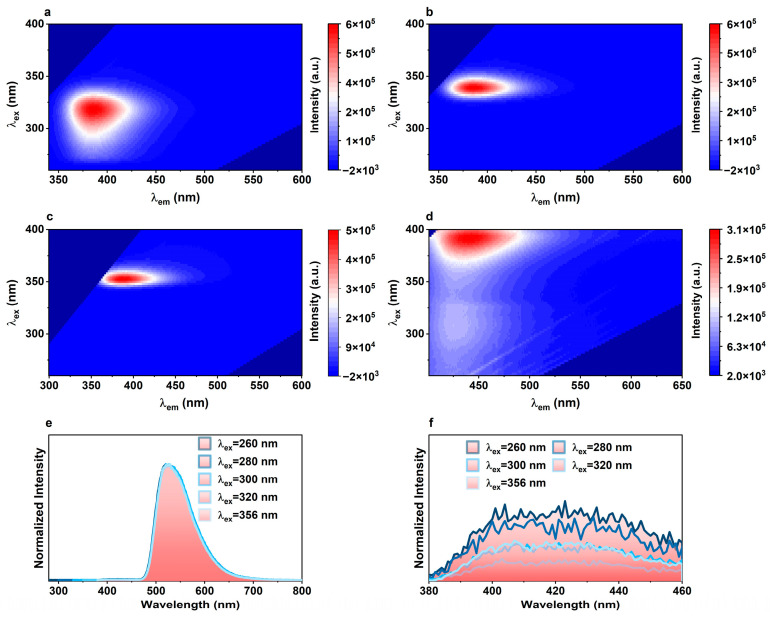
Three-dimensional PL spectra of (**a**) 10^−4^ M, (**b**) 10^−3^ M, and (**c**) 10^−2^ M H_4_L solutions and (**d**) H_4_L in solid state. Phosphorescence spectra of H_4_L (**e**) in solid state at 77 K and (**f**) in range of 380–460 nm.

As reported in prior research, the Δ*E_T_* of TbL is only 161 cm^−1^, which contradicts the empirical rule. To investigate the excited states of the ligand, we examined the luminescence properties of the ligand. Concentration-dependent 3D photoluminescence (PL) spectra of H_4_L were recorded at room temperature and identified as ligand monomer emissions. As depicted in Figure 2a–d, the maximum excitation wavelengths of the H_4_L ligand in solutions shift from 318 nm to 353 nm as the concentrations increase from 10^−4^ to 10^−2^ M, while the emission peaks remain at 387 nm. The *S*_1_ state of H_4_L molecules is thus determined to be 27,400 cm^−1^. For solid H_4_L, the emission maximum redshifts to 442 nm and the excitation maximum to 392 nm, likely due to molecular packing effects.

Next, phosphorescence spectra of the ligand were recorded at 77 K in the solid state upon excitation from 260 nm to 365 nm (Figure 2e,f). The band at 526 nm was assigned to *T*_1_, and the weak band around 420 nm, ~23,800 cm^−1^, was attributed to the ligand’s *T_n_* state. The energy difference between the *T_n_* state and the emitting level ^5^D_4_ is approximately 3300 cm^−1^, suggesting that the *T_n_*→^5^D_4_ energy transfer might also contribute to the observed bright TbL emission. Therefore, we speculate that TbL emission may result from *S*_1_ and higher triplet energy levels of the ligand. A recent example of *S*_1_→Ln^3+^ energy transfer was reported by Jérôme Long and Luís D. Carlos et al., in which the intramolecular energy transfer rates of [Ln(bpy)_2_(NO_3_)_3_] (bpy—2,2′-bipyridine; Ln—Tb or Eu) [26] were determined. At temperatures above 125 K, the *S*_1_→Eu^3+^ channel dominates the sensitization of the ^5^D_0_ level.

### 2.3. Temperature-Dependent Photoluminescence Properties of TbL and Eu_x_Tb_1−x_L

**Figure 3:**
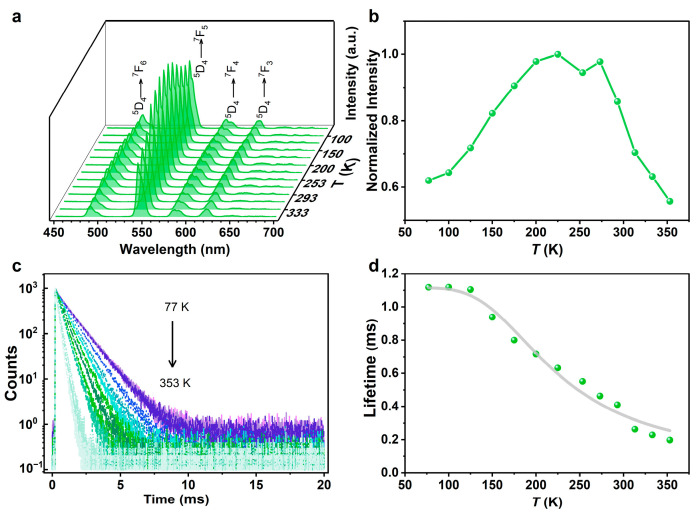
(**a**) Temperature-dependent PL spectra and (**b**) integrated intensities of ^5^D_4_→^7^F_5_ transition of TbL. (**c**) Temperature-dependent ^5^D_4_ decay curves. (**d**) Experimental temperature-dependent ^5^D_4_ lifetime values (green scatters) and the fitted curve (grey line, *R*^2^ = 0.98) of TbL.

To rationalize the energy diagram of this system, we consider the energy transfer from the ligand to Tb^3+^ and the backward energy transfer from Tb^3+^ to the ligand in relation to the temperature-dependent ^5^D_4_ emissions and lifetime. Upon excitation at 351 nm, TbL shows typical emissions around 488, 542, 583, and 621 nm, corresponding to the ^5^D_4_→^7^F_6–3_ transitions (Figure 3a). The relative intensities of the ^5^D_4_→^7^F_5_ (*I*_Tb_) transitions were quantified by integrating the emission spectra between 530 and 570 nm. The *I*_Tb_ exhibited negative thermal quenching in the range of 77–225 K, with a 63% increase, and mild thermal quenching in the range of 22–353 K, with an ≈42% decrease (Figure 3b). The temperature sensitivity of TbL is much lower than that of (Me_2_NH_2_)_3_[Ln_3_(FDC)_4_(NO_3_)_4_]·4H_2_O (H_2_FDC = 9-fluorenone-2,7-dicarboxylic acid) [36], with a prior sample showing low Δ*E_T_*, suggesting a low backward energy transfer rate for TbL. As the first Δ*E_T_*-driven single-lanthanide organic framework ratiometric luminescent thermometer, (Me_2_NH_2_)_3_[Ln_3_(FDC)_4_(NO_3_)_4_]·4H_2_O showed extreme Eu^3+^ emission thermal quenching with increasing temperature. This result aligns with the small energy difference (553 cm^−1^) between the H_2_FDC triplet excited state (17,794 cm^−1^) and the ^5^D_0_ Eu^3+^ level (17,241 cm^−1^), indicating a strong thermally activated ion-to-ligand backward energy transfer. We obtained the activation energies for the nonradiative channels of (Me_2_NH_2_)_3_[Ln_3_(FDC)_4_(NO_3_)_4_]·4H_2_O and found that the values match the experimentally observed energy difference, proving that ion-to-ligand backward energy transfer is the dominant pathway for Eu^3+^ emission thermal quenching. These results suggest that the empirical observations overestimate the contribution of *T*_1_ level to energy transfer between ligands and Ln^3+^ in certain Ln-MOFs.

Figure 3c shows the temperature-dependent ^5^D_4_ decay curves of TbL, which can only be accurately represented by biexponential decay functions, possibly due to the presence of two distinct Tb^3+^ local sites. The determined lifetimes range from 1118 to 198 μs between 77 and 353 K. As it is beyond the scope of the present paper to solve the energy transfer rate and backward energy transfer rate between the *S*_1_, *T_n_*, *T*_1_, and ^5^D_4_ levels, we adopted the empirical Mott–Seitz model here.

(1) $$\tau (T)=\frac{\tau_{0}}{1+\alpha \cdot exp(\frac{-∆E}{k_{B}T})}$$

We used the empirical Mott–Seitz model to fit the temperature-dependent ^5^D_4_ lifetimes (Figure 3d) in the 77–353 K range with *R*^2^ = 0.98. Here, *τ*_0_ represents the lifetimes of the Tb^3+^ local sites at *T* = 0 K, *α* is the ratio between the nonradiative (*T* = 0 K) and radiative rates, Δ*E* represents the activation energy for the nonradiative channels of Tb^3+^, *k_B_* is the Boltzmann constant, and *T* is the absolute temperature. *τ*_0_, *α*, and Δ*E* are determined to be 1115 μs, 36.8, and ≈500–600 cm^−1^, respectively. Comparing the energy barrier value extracted from the phosphorescence spectrum (161 cm^−1^) with that resulting from the Mott–Seitz analysis, we observed a factor of ≈3 between them. This discrepancy might have arisen because of (i) the presence of forward and backward energy transfer between the *S*_1_ or higher triplet energy levels of the ligand and Tb^3+^ in TbL and (ii) the fact that the Mott–Seitz model generally overestimates the energy barrier value.

According to the results, it is reasonable to assume that the *S*_1_ and *T_n_* are involved in the luminescence of Eu_x_Tb_1−x_L as much as the *T*_1_ state is. Thus, exploring Eu_x_Tb_1−x_L could provide a good platform to construct luminescent thermometers with tunable relative thermal sensitivity and operating temperature ranges and to study the contributions of multiple energy transfers and Eu^3+^ content to the temperature-sensing performance of Eu/Tb-MOF thermometers. The room temperature photoluminescence (PL) excitation spectra of Eu_x_Tb_1−x_L were monitored at the ^5^D_4_→^7^F_5_ (Tb^3+^) transition (Figure S3a, Supporting Information), and all excitation spectra were dominated by a broad band around 340 nm, attributed to the ligand’s singlet excited state. The emission spectra of Eu_x_Tb_1−x_L (77–353 K) consisted of characteristic ^5^D_4_→^7^F_6–3_ transitions of Tb^3+^ and ^5^D_0_→^7^F_0−4_ transitions of Eu^3+^ (see Figure S3b). The relative intensities of the ^5^D_4_→^7^F_5_ (*I*_Tb_) and ^5^D_0_→^7^F_2_ (*I*_Eu_) transitions were quantified by integrating the emission spectra between 530–570 nm and 605–640 nm, respectively. To determine absolute temperature, *I*_Tb_ serves as the temperature probe while *I*_Eu_ acts as the reference due to their strong PL intensities, thus enabling precise temperature determination.

**Figure 4:**
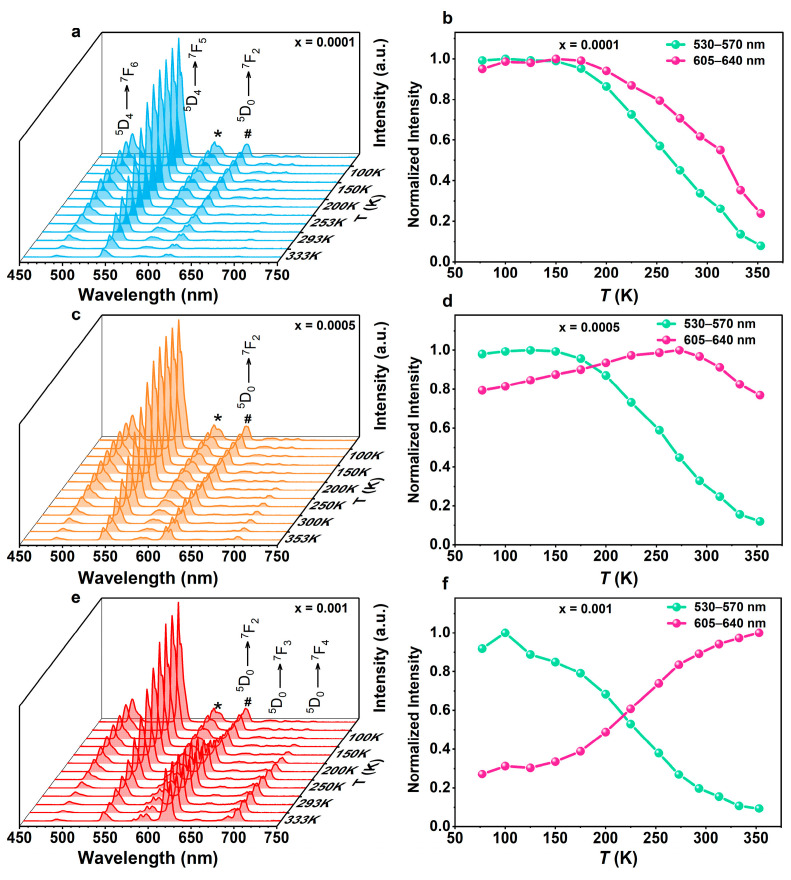
(**a**) Temperature-dependent PL spectra and (**b**) integrated areas of *I*_Tb_ and *I*_Eu_ of Eu_x_Tb_1−x_L (x = 0.0001) upon excitation at 345 nm. (**c**) Temperature-dependent PL spectra and (**d**) integrated areas of *I*_Tb_ and *I*_Eu_ of Eu_x_Tb_1−x_L (x = 0.0005) upon excitation at 345 nm. (**e**) Temperature-dependent PL spectra and (**f**) integrated areas of *I*_Tb_ and *I*_Eu_ of Eu_x_Tb_1−x_L (x = 0.001) upon excitation at 345 nm. Asterisk represents the overlap between Eu^3+ 5^D_0_→^7^F_2_ and Tb^3+ 5^D_4_→^7^F_3_ transitions, and pound sign represents that between Eu^3+ 5^D_0_→^7^F_0,1_ and Tb^3+ 5^D_4_→^7^F_4_ transitions.

For the Eu_x_Tb_1−x_L samples, while the Tb^3+^ emissions followed typical Mott–Seitz model curves across the entire temperature range, the Eu^3+^ emissions displayed very distinct temperature dependence. For the sample with x = 0.0001 (Figure 4a,b), the Tb^3+^ and Eu^3+^ emissions remained relatively constant in the range of 77–175 K, followed by decreases of approximately 92% and 75% up to 353 K, respectively. For the sample with higher Eu^3+^ content (x = 0.0005), the Tb^3+^ emissions underwent typical thermal quenching, decreasing by approximately 88% up to 353 K. Meanwhile, the Eu^3+^ emissions displayed negative thermal quenching in the range of 77–253 K (Figure 4c,d), with an increase of approximately 26% followed by a decrease of approximately 23% up to 353 K. With increasing Eu^3+^ content (x = 0.001), the Eu^3+^ emissions showed stronger negative thermal quenching, rising by ≈270% up to 353 K (Figure 4e,f), while the Tb^3+^ emissions decreased by ≈90% up to 353 K. This indicates that the thermal sensitivity of Eu^3+^ emissions progressively increases with rising Eu^3+^ content, making Eu_x_Tb_1−x_L excellent candidates for ratiometric thermometers with tunable thermometric performances.

**Figure 5:**
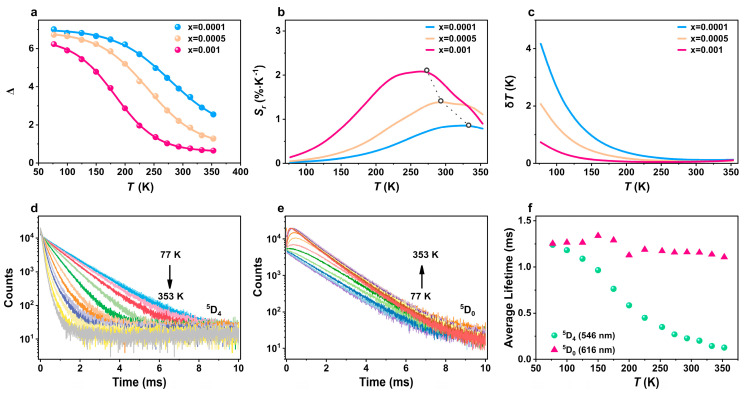
(**a**) Thermometric parameters, (**b**) relative thermal sensitivities, and (**c**) temperature uncertainties of Eu_x_Tb_1−x_L samples within the temperature range of 77–353 K. Temperature-dependent PL decay curves of (**d**) Tb^3+^ and (**e**) Eu^3+^ and (**f**) temperature-dependent PL lifetimes for the sample with x = 0.001 in the 77–353 K range.

Here, the ratio of ^5^D_4_→^7^F_5_ transition to *I*_Eu_ was used to define the thermometric parameter Δ = *I*_Tb_/*I*_Eu_. We note that while *I*_Tb_ corresponds only to the ^5^D_4_→^7^F_5_ transition, the *I*_Eu_ integration range includes a small contribution from the Tb^3+ 5^D_0_→^7^F_3_ transition. Nevertheless, all the thermometric parameters of the Eu_x_Tb_1−x_L samples were fitted to an empirical sigmoidal Boltzmann function,

(2) $$∆=\frac{I\left(Tb\right)}{I\left(Eu\right)}=\frac{A_{1}-A_{2}}{1+e^{\frac{T-T_{0}}{-dT}}}+A_{2}$$

where *I*(Tb) is the emission intensity in the wavelength range of 530–570 nm, *I*(Eu) is the emission intensity in the wavelength range of 605–640 nm, *A*_1_ represents the maximum emission intensity and *A*_2_ represents the minimum emission intensity, *T*_0_ is the temperature at which the emission intensity reaches half of *A*_1_, and *T* is the absolute temperature. The fit results show *R*^2^ > 0.999, implying that the thermometer performance was unaffected by the presence of the ^5^D_0_→^7^F_3_ transition. This holds true for all the Eu^3+^ contents studied in this study. Therefore, in the following analysis, we considered the commonly assumed labeling of *I_Eu_* as being solely due to the ^5^D_0_→^7^F_2_ contribution. Figure 5a displays the calibration curves of Eu_x_Tb_1−x_L samples within the temperature range of 77–353 K under 335 nm excitation. The fitting parameters for the Eu_x_Tb_1−x_L compounds are listed in Table 1 and show a nearly constant Δ_0_ and an increase in *T*_0_ as the Eu^3+^ content increased. By increasing the amount of Eu^3+^ from x = 0.0001 to 0.001, a shift of 95 K toward lower temperatures was observed in *T*_0_.

The relative sensitivity was employed to compare the performance of this model with those of similar thermometers reported in previous studies, defined as

(3) $$S_{r}=\frac{1}{∆}\cdot \left|\frac{\partial ∆}{\partial T}\right|$$

The maximum relative temperature sensitivities of the thermometers were 0.86% K^−1^ at 333 K, 1.42% K^−1^ at 293 K, and 2.11% K^−1^ at 273 K for, respectively, the samples with x from 0.0001 to 0.001 (Figure 5b). Intriguingly, in the physiological range, the relative sensitivities were still above 1.3% K^−1^ for the sample with x = 0.0005 and 1.5% K^−1^ for the sample with x = 0.001, while the corresponding temperature uncertainties were estimated from

(4) $$\delta T=\frac{1}{S_{r}}\cdot \frac{\delta ∆}{∆}$$

yielding the values of 0.08 K for the sample with x = 0.0005 and 0.07 K for the sample with x = 0.001 at 313 K (Figure 5c). This variation in the *S_r_* is partially explained by the increase in the nonradiative decay rate of the Tb^3+ 5^D_4_→^7^F_5_ transition (relatively to the radiative one) with the increase in Eu^3+^ doping.

### 2.4. Energy Transfer

To rationalize the thermal dependence of the luminescence of the materials, we further analyzed the energy transfer from Tb^3+^ ions to Eu^3+^ ions using the sample with x = 0.001 as an example. Generally, the long distances between these ions may result in low rates that cannot compete with the ligand-to-Ln^3+^ rates, which are orders of magnitude higher. The thermal dependence of the Tb^3+^ and Eu^3+^ lifetimes was investigated by monitoring the emission decay curves of the ^5^D_4_→^7^F_5_ and ^5^D_0_→^7^F_2_ transitions (Figure 5d,e), respectively. All the decay curves are well modeled and show that the ^5^D_0_ lifetime remains stable up to 1289 μs at 175 K, slightly decreasing to 1104 μs at 353 K. As the temperature rose from 77 K to 353 K, the ^5^D_4_ lifetime progressively decreased from 1239 μs to 127 μs, remaining nearly independent of the Eu^3+^ doping until *T* > 175 K (Figure 5f). Notably, a rise time, dependent on the temperature, occurred in the ^5^D_0_ emission decay curves in the temperature range from 175 K to 353 K (Figure 5e). This rise time was also observed in [Ln(bpy)_2_(NO_3_)_3_] by Jérôme Long and Luís D. Carlos et al. [26] and was found to be similar to the ^5^D_4_ lifetime. This implies that the ^5^D_4_ level could support Tb^3+^-Eu^3+^ energy transfer within Eu_0.001_Tb_0.999_L above 175 K.

## 3. Materials and Methods

### 3.1. Materials and Characterization

All chemicals used in this work were commercially available and used without further purification. The X-ray powder diffraction (PXRD) patterns were collected using a D/MAX 2500/PC powder diffractometer (Rigaku, Tokyo, Japan) equipped with a Cu Kα radiation source, covering a 2*θ* range of 5–50°. The FT-IR spectra of samples embedded in KBr pellets were recorded using a PerkinElmer FT-IR spectrometer. Thermogravimetric analysis (TGA) was conducted on a TG/DTA 6300 thermal gravimetric analyzer (Hitachi, Tokyo, Japan) at a constant rate of 10 K/min. Room temperature luminescence spectra were obtained using an FS5 steady-state transient fluorescence spectrometer with a 150 W CW ozone-free xenon lamp. Temperature-dependent luminescence spectra were collected using an FLS1000 photoluminescence spectrometer (Edinburgh Instruments Ltd., Livingston, UK) with a 300 W CW ozone-free xenon lamp.

### 3.2. Preparation of [Tb_2_L_1.5_(NMP)_2_]_n_ (TbL)

A solution of H_4_L was prepared by dissolving H_4_L (0.2 mmol, 81.2 mg) in 24 mL of N-methyl-2-pyrrolidone (NMP), and a Tb(NO_3_)_3_ solution was prepared by dissolving Tb(NO_3_)_3_·6H_2_O (0.4 mmol, 180.5 mg) in 24 mL of H_2_O. These two solutions were then mixed in a 100 mL Teflon-lined stainless-steel autoclave, followed by the addition of 200 μL of concentrated hydrochloric acid. The mixture was sealed and maintained at 433 K for 3 days. After the autoclave was gradually cooled to room temperature, colorless rhombic crystals of TbL (Yield: 65%) were obtained by filtration after washing with ethanol three times.

### 3.3. Preparation of Eu_x_Tb_1−x_L (x = 0.0001, 0.0005, and 0.001)

Eu_x_Tb_1−x_L (x = 0.0001, 0.0005, and 0.001) samples were prepared using a method similar to that used for TbL, with the exception that the Tb(NO_3_)_3_·6H_2_O solution was replaced with a mixture of Eu(NO_3_)_3_·6H_2_O (x⋅24 mL) and Tb(NO_3_)_3_·6H_2_O (24(1−x) mL) solutions.

## 4. Conclusions

This study offers a thorough analysis and explanation of the luminescence mechanisms of TbL and the fine-tuning of the temperature sensitivity of Eu_x_Tb_1−x_L thermometers. Different from the luminescence mechanisms of similar mixed Eu/Tb-MOFs, the *S*_1_ and higher triplet energy levels of the ligand are involved in the sensitization of Tb^3+^ emission. Therefore, the judicious choice of ligand and Eu^3+^ content makes Eu_x_Tb_1−x_L excellent thermometers, with a relative thermal sensitivity comparable to those of similar mixed Eu/Tb-MOF-based luminescent thermometers operating across a temperature range from cryogenic to physiological. Additionally, the analysis of the luminescence lifetimes of the sample with x = 0.001 provides a clear understanding of the temperature dependence of the luminescence spectra. Furthermore, this approach serves as a platform that can be leveraged to guide the design of new Eu/Tb thermometers based on dual-emissive centers. The results enable lots of neglected similar materials to show luminescent temperature-sensing properties. Notably, since the precise prediction of multiple-energy-level-involved luminescence is complex within mixed Eu/Tb-MOFs, it is still a challenge to design similar materials.

## Figures and Tables

**Table 1:** Fitting results of Δ *(T)* to Equation (2).

Items	*A*_1_	*A*_2_	*T*_0_	*R* ^2^
Eu_0.0001_Tb_0.9999_L	6.70	1.20	278.00	0.9990
Eu_0.005_Tb_0.999_ L	6.80	0.42	240.00	0.9993
Eu_0.001_Tb_0.999_L	6.40	0.10	183.00	0.9997

## Data Availability

All data generated or analyzed during this study are included in this published article and its Supplementary Information Files.

## Supplementary Materials

The following supporting information can be downloaded at: https://www.mdpi.com/article/10.3390/molecules29163914/s1, including the data for other structural characterizations, powder XRD patterns, IR spectra, TGA curves and PL spectra. Figure S1: PXRD patterns of TbL and Eu_x_Tb_1−x_L (x = 0.0001, 0.0005, and 0.001). Figure S2: Thermal gravimetric curves of TbL and Eu_x_Tb_1−x_L (x = 0.0001, 0.0005, and 0.001). Figure S3: The IR spectra of TbL and Eu_x_Tb_1−x_L (x = 0.0001, 0.0005, and 0.001). Figure S4: Excitation spectra and emission spectra of Eu_x_Tb_1−x_L (x = 0.0001, 0.0005, and 0.001).
