# Supplementary material for: Tailoring Energy Transfer in Mixed Eu/Tb Metal–Organic Frameworks for Ratiometric Temperature Sensing
Source: Molecules. 2024 Aug 19;29(16):3914. doi: 10.3390/molecules29163914 (PMC11357012; doi:10.3390/molecules29163914)
Supplement: Supplementary file 1 [file molecules-29-03914-s001.zip › molecules-3131611-supplementary.pdf]

Supporting Information

Tailoring Energy Transfer in Mixed Eu^{3+}/Tb^{3+} Metal–Organic Frameworks for Ratiometric Temperature Sensing

Hui Tang [1], Siyuan Cheng [1], Zhihui Zhang [1,*], Mingyang He [1], Junfeng Qian [1], Liang Li [2,*]

[1] Jiangsu Key Laboratory of Advanced Catalytic Materials and Technology, Changzhou University, Changzhou, 213164, P. R. China.

[2] Xinjiang Key Laboratory for Luminescence Minerals and Optical Functional Materials, School of Physics and Electronic Engineering, Xinjiang Normal University, Urumqi, Xinjiang 830054, P. R. China.

* Correspondence: zhangzh@cczu.edu.cn (Z.Z.); speeliangl@xjnu.edu.cn (L.L.)

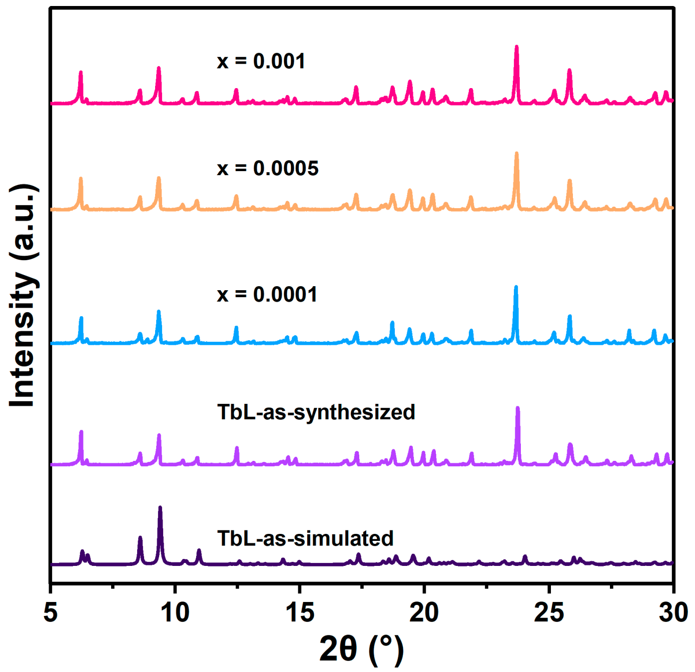

Figure S1. PXRD patterns of **TbL** and **$Eu_xTb_{1-x}L$ (x = 0.0001, 0.0005, and 0.001).**

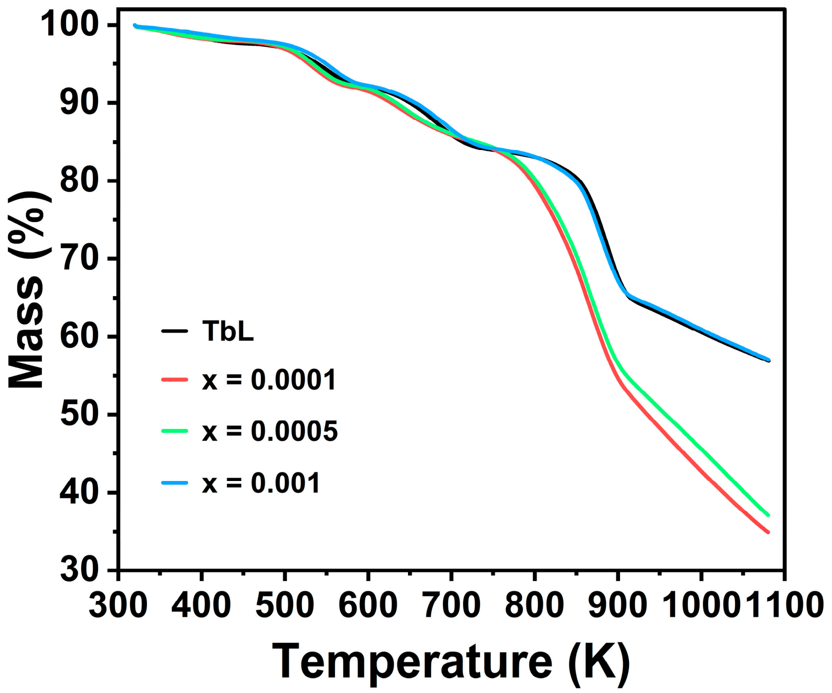

Figure S2. Thermal gravimetric curves of **TbL** and **Eu$_x$Tb$_{1-x}$L (x = 0.0001, 0.0005, and 0.001).**

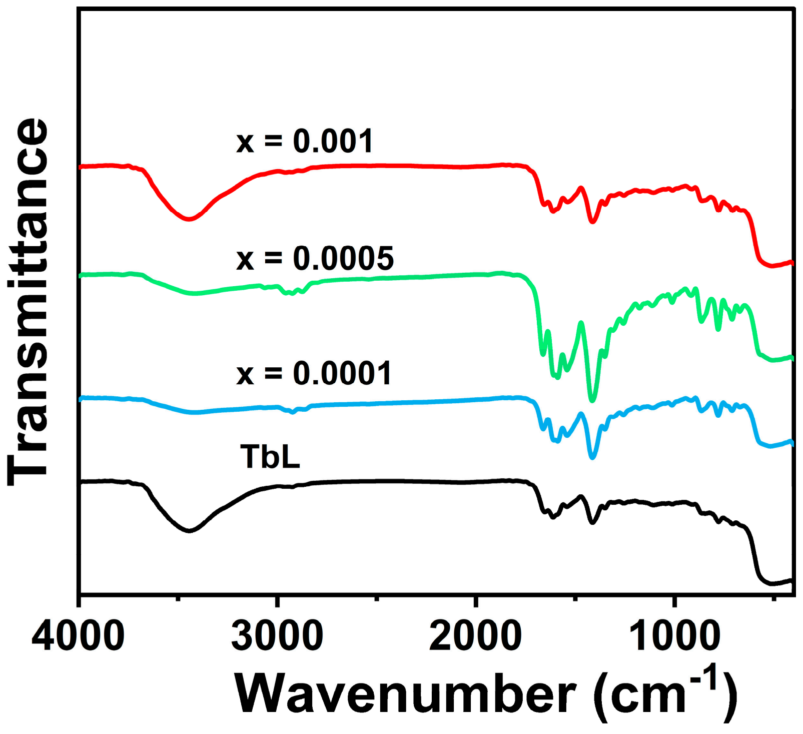

Figure S3. The IR spectra of **TbL** and **Eu$_x$Tb$_{1-x}$L (x = 0.0001, 0.0005, and 0.001)**

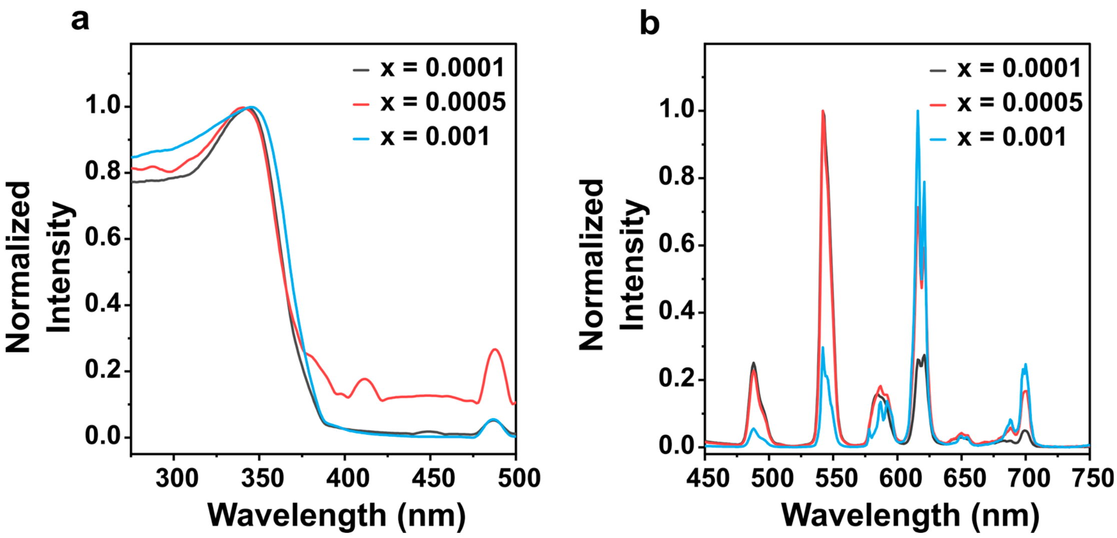

Figure S4. (a) Excitation spectra and (b) emission spectra of **$Eu_xTb_{1-x}L$ (x = 0.0001, 0.0005, and 0.001)** at room temperature.
